# The Relations between Father-Perceived Family Strength and Maternal Gatekeeping in Chinese Families—Focusing on the Dual Mediation Effect of Father Involvement and Mutual Communication

**DOI:** 10.3390/bs13120968

**Published:** 2023-11-24

**Authors:** Shu Zhang, Hae-Shin Hwang

**Affiliations:** 1 Department of Family Welfare, Sangmyung University, Seoul 04763, Republic of Korea; 2 West Campus, Shandong University of Technology, Zibo 255000, China

**Keywords:** maternal gatekeeping, mutual communication, father involvement, psychological stress, family strength

## Abstract

According to Murray Bowen’s family systems theory, the family is an emotional unit where members influence each other. Family strength, in that members care for, respect, and communicate with each other sincerely and freely and overcome difficulties together through joint efforts, is what every family yearns for and pursues. Maternal gatekeeping behavior not only affects the relationship between each family member but also has an impact on family strength. Father-perceived family strength and maternal gatekeeping were investigated in this study, along with the mediation effect of father involvement and mutual communication. In total, 320 fathers of children aged 3–5 were randomly recruited to complete self-report questionnaires. The correlation results indicated that maternal gate-opening behavior, father involvement, mutual communication, and family strength are positively correlated with each other, whereas maternal gate closing is negatively associated with father involvement, mutual communication, and family strength. Path analysis verified the partial (dual) mediation effect of father involvement and mutual communication between maternal gate opening and family strength, as well as a complete (dual) mediation effect upon maternal gate closing. The current study provides new insights into understanding the underlying mechanisms of father-perceived family strength influenced by maternal gatekeeping behavior. Meanwhile, the mediation effect of father involvement and mutual communication also highlights the importance of parenting preparation for fathers, such as psychological self-adjustment, especially under interception or restriction.

## 1. Introduction

Family is the basic unit and form of society, and the strength of the family is the core of a healthy society [1]. Family strength has been studied by various scholars, among whom Defrain defined that in a strong family, all members should have satisfactory interactions in all sub-relationships, and their development should also be promoted [2,3]. In this study, based on a study by Xu Anqi, family strength is defined as a harmonious relationship among family members, characterized by mutual care, respect, open and free communication, and the ability to work together to solve problems [4]. Family is not only the basic environment for individuals to experience but also the starting point of individual socialization, especially those with children aged 3–5. When family strength is reliable enough, individuals can realize their full potential. Conversely, unhealthy family relationships or poor family functioning can lead to serious problems, such as problematic behavior, maladjustment, and even crime [5]. Members in strong families can cooperate and help each other, and each family member can be trusted and enjoy their inner peace and happiness. In other words, a family with good strength is an important guarantee and support for promoting the healthy growth of family members and bringing them together to overcome difficulties in the face of adversity [6].

### 1.1. The Direct Effect of Maternal Gatekeeping on Father Involvement and Family Strength and the Mediation Effect of Father Involvement

With the development of society and the advancement of urbanization in China, family sizes in China have decreased significantly, and fathers are now required to take on more responsibilities, including child-rearing. As raising children together is becoming an inevitable trend, the father’s role becomes increasingly important. However, not all mothers have confidence in or support the father. Some mothers are viewed as goalkeepers on the football field, striving to prevent the opposing team from scoring. Fagan J. defined mother gatekeeping as mothers’ preference for handling matters related to their children independently and attempting to restrict and reject fathers’ care and participation in their children’s activities [6]. Notably, Austin, W. G. and coworkers expanded the understanding of mother gatekeeping, defining it as a continuous spectrum ranging from hindrance behavior to support behavior. This spectrum includes two categories, i.e., opening behavior and closing behavior [7]. Moreover, it has been highlighted that restriction and promotion are not two opposite poles of a continuous spectrum and that they can co-exist [8]. Through the crossover hypothesis based on family systems theory (the emotional or behavioral impact of one member in the subsystem can affect the behavior of other members), it can be predicted that the conscious or unconscious gatekeeping behavior of mothers can directly affect the level of father involvement in parenting. Schoppe-Sullivan and coworkers’ study shows that father involvement and competence in caring for their infants may be most susceptible to maternal gatekeeping and may lay the ground work for the high-quality father–child relationships that have been consistently linked to the positive development of children [9]. In other words, maternal emotional or behavioral support or interference in the parental subsystem might influence the father’s attitude and behavior towards parenting their children in the father–child subsystem. It reflects the direct effect of gatekeeping behavior on father involvement, and the importance of father-perceived gatekeeping behavior on children’s development has also been clarified. This not only affects the father but also has an impact on the relationships among family members, which in turn affects family strength. Liu et al. highlight the significant effect of father involvement in child-rearing on the strength of dual-income families; this not only reflects gender equality but also enhances marital relationships and positively affects family strength through effective joint parenting [10].

### 1.2. The Direct Effect of Maternal Gatekeeping on Mutual Communication and the Mediation Effect of Mutual Communication

Gatekeeping behavior can also affect marital relationships. The spillover hypothesis suggests that the emotions, actions, and atmosphere generated by one subsystem of a family can be transferred to other subsystems [11]. For instance, in the parenting subsystem, mothers’ gatekeeping behavior can facilitate or impede parental co-parenting. It can either enhance or disrupt mutual communication between couples in the marital relationship subsystem. Marital relationships are paramount in families, and communication, being a relational factor, serves as the foundation for interaction between couples and significantly contributes to marital satisfaction [12]. The satisfaction of marriage declines when couples have minimal or negative communication, leading to significant strain in their marital relationships due to inefficient communication. Conversely, couples who engage in clear, open, and efficient information exchange can sustain a contented and robust marriage [13]. In addition, increased communication among couples is associated with greater family strength [14]. This suggests that mutual communication between partners is a significant factor in family strength. Therefore, maternal gatekeeping behavior has a direct effect on mutual communication, and mutual communication plays a mediating role between maternal gatekeeping behavior and family strength.

### 1.3. The Interaction between Father Involvement and Mutual Communication

At the same time, father involvement and mutual communication are mutually influential. Regarding marital relationships during the child-rearing period, there is a significant change compared to the early marriage stage. This is a time when marital satisfaction plummets due to stress caused by child-rearing issues. Choi believes that, during this phase, the active involvement of fathers in parenting not only impacts children but also affects mothers and marital relationships [15]. Mothers who experience parenting as a burden can benefit from their husbands’ active involvement in parenting. When a mother perceives her husband as a supportive partner, it positively impacts her psychological well-being. Consequently, enhancing father involvement can enhance marital relationships and mutual understanding, leading to improved mutual communication. Kim found that higher marital satisfaction, including effective communication between partners, is directly linked to increased father involvement [16]. Conversely, based on the research of Chung et al., when insufficient communication arises in a marital relationship, father involvement in parenting tends to diminish [17].

### 1.4. The Current Study

**Figure 1:**
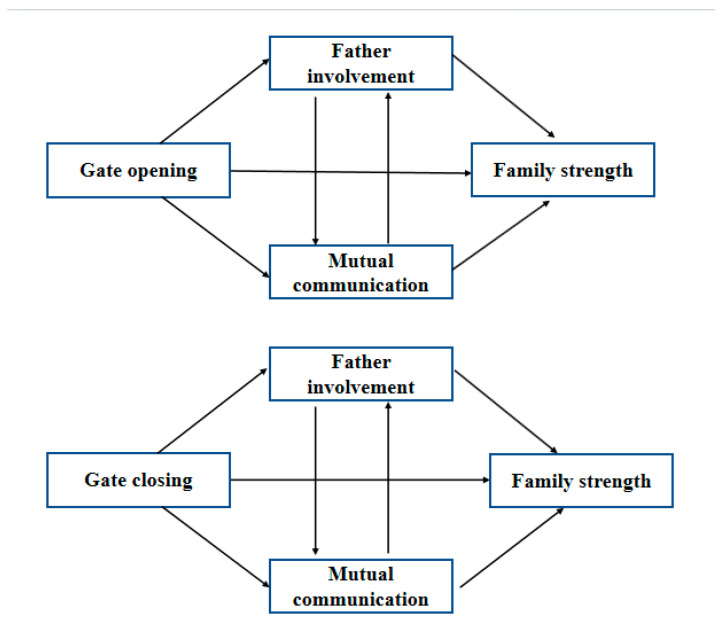
Research model.

Based on C. Gaffney’s research, self-assessments are to some extent inaccurate [18]. Moreover, in D. Dunning’s psychological and educational theory, there is a tenuous to modest relationship between people’s self-view and their actual behavior and performance [19]. As primary caregivers, mothers may exhibit biases in their self-evaluation. Thus, evaluating gatekeeping behavior and family status from the father’s viewpoint has high reference value. Up to now, there is no research related to the influence of father-perceived gatekeeping behavior on family strength, including its effect on father involvement and mutual communication. Thus, in this work, considering the importance of father involvement in child rearing, the relationship was systematically investigated among 320 fathers who have children aged 3–5 years, and the mediating effects of father involvement and mutual communication were also systematically analyzed. A research model was established and is shown in Figure 1.

This research can effectively avoid the deviation caused by the mother’s self-assessment or the researchers’ observations. With the relationship between father-perceived family strength and maternal gatekeeping systematically investigated, the partial (dual) and complete (dual) mediation effects of father involvement and mutual communication are revealed in detail under various gatekeeping situations.

## 2. Materials and Methods

### 2.1. Participants

The participants had to be fathers of children aged 3–5 years old. Both biological parents had to be present in the family. The family had to be intact, without separation, divorce, or preparations for divorce. Moreover, the participants had to have no history of domestic violence or abuse. The general characteristics of the participants can be found in Table 1. Based on Shin and Chung’s study, variables such as monthly income and educational level can affect family strength [20]. In terms of GDP ranking among the 283 cities in China, Zibo city is positioned at 65th place, while Binzhou city ranks 110th. Thus, taking the demographic and socioeconomic aspects into account, we chose participants from Zibo and Binzhou in Shandong province, which exhibits a moderate level of development in China, as the subjects of our survey. A preliminary survey was conducted on 30 fathers with 3–5-year-old children enrolled in a kindergarten in Zibo and Binzhou, Shandong province, to determine the appropriateness of the questionnaire contents used in this survey and the time required for response. A preliminary survey was conducted with cooperation from the kindergarten by sending a questionnaire to the fathers through email. Based on the preliminary findings, the feasibility and understandability of the questionnaire content are not problematic. The content of the questionnaire effectively fulfills the objective of data collection, making it suitable for this study. The estimated time required for completing the questionnaire survey is approximately 9 min.

An informed consent form was sent to every child’s father before this study. A total of 344 fathers of infants aged three to five were sent questionnaires (shown in Supplementary Materials File S1), with their signed consent given. A total of 320 copies were used for the final analysis, excluding 24 copies with dishonest answers (all answers for each question are the same), from single-parent families, or those with a response time of less than 200 s. The time limit of 200 s is mainly due to the fact that during the pre-survey, through on-site timing, the shortest time for serious completion is more than 200 s. Therefore, questionnaires completed in less than 200 s were suspected of not being filled out seriously. In order to ensure the reliability of the data, questionnaires completed in less than 200 s needed to be excluded, with an effective recovery rate of 93%.

### 2.2. Research Instruments

#### 2.2.1. Family Strength

Family strength is related to the health level of families. To measure the family strength perceived by the father, the Chinese Family Strength Scale revised by Xu [21] based on the Family Adaptability and Cohesion Evaluation Scale of Olson et al. [22] and the Family Environment Scale (FES) of Moos and coworkers [23] was used. It is believed that a harmonious family can not only promote the development of family members, but also enhance the intimate interaction between family members, and it has the flexibility and adaptability to overcome crises. This scale contains a total of 23 questions, comprising seven questions of “dedication and care”, seven questions of “family resilience”, five questions of “mutual respect and tolerance”, and four questions of “love expression and share”. Each question was measured on a five-point Likert scale. The correlation between family strength and the measured score is such that a higher score corresponds to better family strength. The Cronbach’s α value for the overall family strength is 0.834, with the sub-factors exhibiting values of 0.857, 0.871, 0.786, and 0.843, respectively.

#### 2.2.2. Maternal Gatekeeping

Maternal gatekeeping can be defined as a mother’s behavior aimed at controlling the degree of the father’s participation in child parenting. To analyze the maternal gatekeeping perceived by the father, the gatekeeping behavior measurement scale supplemented by Sullivan et al. [24] and finally modified by Yee and coworkers was applied in the research [25]. It comprises two sub-factors (gate opening and gate closing) and a total of 17 questions. Each question is measured with a six-point Likert scale, in which a large score indicates a high level of maternal gatekeeping perceived by the father. The Cronbach’s α value for maternal gate opening and closing are 0.908 and 0.785, respectively.

#### 2.2.3. Father Involvement

Father involvement is related to the time a father spends with his children and the things carried out for them. The study utilized the Inventory of Father Involvement (IFI), which was developed by Hawkins et al. [26] and translated and revised by Yin et al. [27]. It comprises a total of 18 questions, involving rule guidance, kindergarten life support, mother assistance, childcare support, companion, and encouragement [26]. The question amount, serial number, and Cronbach α values for each sub-factor of the family strength, maternal gatekeeping, and father involvement are presented in Table 2.

#### 2.2.4. Mutual Communication

Mutual communication is defined as a process that affects each person’s behavior while exchanging information and opinions between partners. In this survey, the mutual communication instrument was translated by Bai in 2010 [28] and based on the 10 areas of marriage satisfaction in the ENRICH (Enriching and Nurturing Relationship Issues, Communication, and Happiness) Inventory developed by Olson, Russell, and Sprinkle in 1983 [29]. It utilizes modified couple communication scales and consists of a total of 10 questions with a five-point Likert measure. This tool measures the extent to which couples share their emotions, thoughts, and beliefs through verbal and nonverbal information exchange while understanding each other’s differences; it aims to achieve a satisfactory family life and parenting way. Questions 2, 3, 4, 5, 6, 7, and 9 were scored in reverse. The range of scores that can be obtained is from 10 to 50, with higher scores indicating smoother communication between couples. The Cronbach α value for this tool was found to be 0.673.

The data collected in this study were analyzed using SPSS 25.0 and PROCESS Macro 3.5. The Cronbach’s ɑ coefficient indicates the reliability of the research measurement tools. Furthermore, the frequency, percentage, mean, and standard deviation were calculated using descriptive statistical analysis to find out the demographic characteristics. Additionally, a *t*-test and one-way ANOVA were conducted to find out the difference in family health according to the participants’ demographic and sociological background, with the Scheffé test carried out as a post hoc test [30]. Based on the statistical analysis (Table 3), family strength is not influenced by age, education level, occupation, and number of children but rather by monthly income. To further verify the influence of monthly income on family strength, an analysis was carried out on four groups with different monthly incomes. The M(SD) results are 95.80 (17.64), 93.33 (13.95), 96.55 (11.07), and 98.64 (10.97), which are statistically significant (F = 2.92, *p* < 0.05), and Scheffé verification was conducted as a post-test. It was revealed that, statistically, fathers with monthly incomes exceeding 10,000 RMB perceive a higher level of family strength compared to those with monthly incomes of 3000 to 6000 RMB. In this study, potential confounding factors were eliminated to obtain the net effect between family strength, maternal gatekeeping, father involvement, and mutual communication. The correlation between maternal gatekeeping, father involvement, mutual communication, and family strength was systematically confirmed through Pearson analysis with the mediation effect further verified via a bootstrapping test.

## 3. Results

Compared to most previous research with a single family subsystem, this work paid attention to the family system with three subsystems, i.e., the father–child subsystem, the father–mother subsystem, and the mother–child subsystem. The father–mother subsystem was unveiled to play an intermediation effect between maternal gatekeeping and father’s involvement. The influence of parenting involvement in the father–child subsystem on the father–mother subsystem was also analyzed in this work. It deeply explores the influence of mothers’ gatekeeping behavior on the couple subsystem and father–child subsystem based on family system theory. Family strength covering the overall state of the father, mother, and child was measured as the dependent variable. Previous research mainly emphasizes the impact of the mother’s gatekeeping behavior on the father’s parenting involvement, treating the father as a passive recipient [31].

### 3.1. The Correlation between Father-Perceived Maternal Gatekeeping, Father Involvement, Mutual Communication, and Family Strength

Pearson’s correlation coefficient was calculated to examine the correlation between maternal gatekeeping, family strength, father involvement, and mutual communication, and the results are shown in Table 4, indicating a significant correlation. Specifically, maternal gate opening has a positive correlation with family strength (r = 0.681, *p* < 0.001), whereas maternal gate closing shows a negative influence on family factors (r = −0.171, *p* < 0.01), including dedication and care (r = −0.243, *p* < 0.001), respect and tolerance (r = −0.174, *p* < 0.01), and love and share (r = −0.127, *p* < 0.05). Encouragement and support could promote father involvement during the parenting process, and this is also consistent with the research of Marcell and coworkers [32]. With sufficient encouragement and gate-opening response, fathers generally provide more support and develop greater emotional intimacy with their family members [33]. There is also a positive correlation between family strength and father involvement (r = 0.681, *p* < 0.001). As expected, family strength also has a significant positive correlation with mutual communication (r = 0.502, *p* < 0.001). Notably, the gate-opening behavior of the mother has a significant promotion impact on all sub-factors of father involvement, while gate-closing behavior hinders the father’s parenting participation (r = −0.222, *p* < 0.001). Although gate-closing behavior does not show a significant negative correlation with kindergarten life support, it has a negative correlation with the remaining sub-factors, e.g., rule guidance (r = −0.170, *p* < 0.01), mother assistance (r = −0.235, *p* < 0.001), childcare support (r =−0.239, *p* < 0.001), companion and communication (r =−0.152 *p* < 0.01), and encouragement and praise (r = −0.209, *p* < 0.001). A mother’s gate-closing behavior limits the interaction between the father and child and gradually has a negative impact on child development [34]. Moreover, the mother’s gate-closing behavior in the parent system can to some extent hinder pleasant parenting behavior in the father–child system [35,36]. Moreover, communication between couples has a positive correlation with the mother’s gate-opening role (r = 0.503, *p* < 0.001) and a negative relationship with gate-closing behavior (r = −0.400, *p* < 0.001). Notably, the positive correlation between father involvement and mutual communication was also identified (r = 0.412, *p* < 0.001). 

### 3.2. Mediation Effect of Father Involvement and Mutual Communication between Maternal Gatekeeping and Family Strength

#### 3.2.1. Mediation Analysis upon Maternal Gate Opening

The analysis was conducted using SPSS PROCESS Macro Model 4 to verify the mediation effect of father involvement when the mother plays a gate-opening role, and the results are presented in Table 5. A *t*-test is one type of inferential statistics used to determine whether a significant difference exists between the means of two groups. The SPSS software includes a *t*-test function. This built-in function can take the raw data, calculate the t-value, then compare it to the critical value, and generate the *p*-value. Based on the results, gate-opening behavior has a positive correlation with father involvement (β = 0.81, *p* < 0.001) and family strength (β = 1.15, *p* < 0.001). When the independent and intermediate variables (gate-opening behavior and parenting involvement, respectively) were applied into SPSS PROCESS Macro Model 4, the results indicate that they can effectively promote family strength ((β = 0.92, *p* < 0.001) and (β = 0.27, *p* < 0.001), respectively). Based on the bootstrapping results exhibited in Table 6, there is an apparent mediation effect (β = 0.22, 95% CI = 0.10~0.36). The mother’s gate-opening behavior may also positively influence mutual communication (β = 0.34) and family strength (β = 1.15) (Table 5), and the mediation effect of mutual communication can be further identified in Table 6 (β = 0.18, 95% CI = 0.09~0.29).

#### 3.2.2. Dual Mediation Analysis upon Maternal Gate Opening

**Figure 2:**
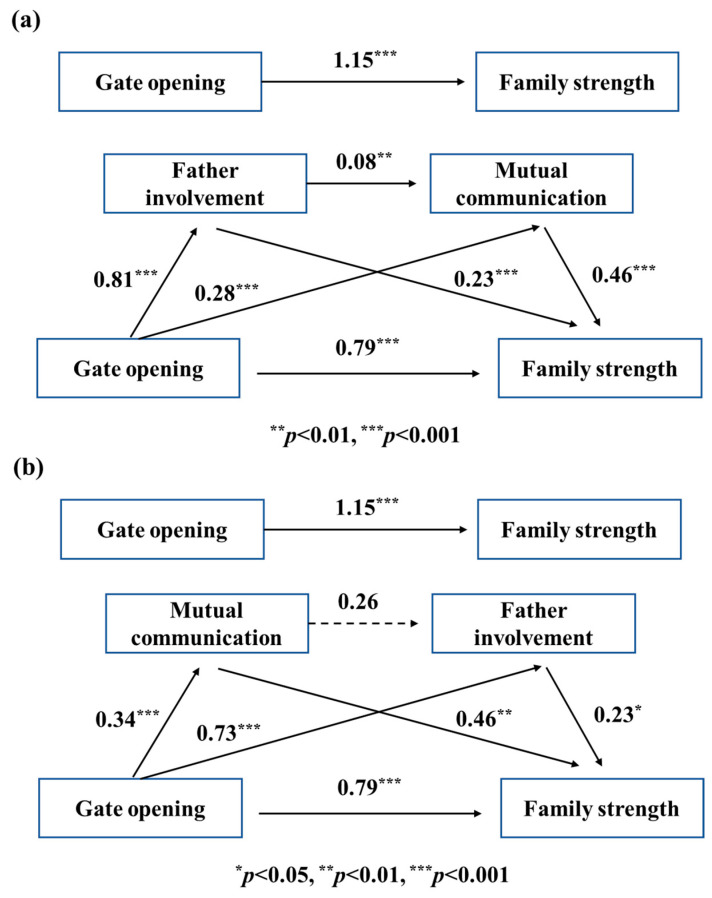
Dual mediation model of (**a**) “father involvement-mutual communication” and (**b**) “mutual communication-father involvement” between maternal gate opening and family strength.

To investigate whether there is a dual mediation effect of father involvement and mutual communication in the relationship between maternal gate-opening and family strength, SPSS PROCESS Macro Model 6 was utilized to analyze the collected data (Table 5). Apparently, both gate-opening behavior (β = 0.28) and father involvement (β = 0.08) can facilitate mutual communication (Figure 2a). With these three parameters applied in the model, there are also beneficial influences on family strength, with them showing β values of 0.79, 0.23, and 0.46, respectively. Thus, the dual intermediation effect of father involvement and mutual communication can be verified based on theory. As for the bootstrapping analysis relating to the pathway involving gate-opening behavior, father involvement and mutual communication, 0 is not in the 95% confidence interval, demonstrating a clear dual mediation effect (β = 0.03, CI = 0.01~0.07) (Table 6).

Nevertheless, based on Figure 2b, there is no clear correlation between mutual communication and father involvement (β = 0.26, *p* > 0.05). The bootstrapping results (Table 6) demonstrate that 0 is involved in the 95% confidence interval of the gate-opening→mutual communication→father involvement→family health pathway (β = 0.02, CI = −0.00~0.06). Thus, statistically, there is no dual intermediate effect.

#### 3.2.3. Mediation Analysis upon Maternal Gate Closing

Based on the results analyzed using SPSS PROCESS Macro Model 4 (Table 7), father-perceived gate-closing behavior has a negative effect on father involvement (β = −0.31, *p* < 0.001) and family strength (β = −0.31, *p* < 0.01). When the mother’s gate-closing behavior perceived by the father and father involvement are both applied in the SPSS model, it indicates no apparent correlation between gate-closing behavior and family health state (β = −0.09, *p* > 0.05). Interestingly, the father’s parenting participation could still significantly promote family strength (β = 0.70, *p* < 0.001). Since the direct influence of gate-closing behavior on family strength could not be detected, there was a complete mediation effect of father involvement, which was further confirmed in the bootstrapping analysis (β = −0.22, 95% CI = −0.35~−0.10), as shown in Table 8. If a father actively joins the parenting process, family strength can still be kept at a high level. Thus, enhancing fathers’ parenting awareness and ability is of high value for the stability of families and the development of children. A father with high-level resilience can eliminate the negative effects of disapproval or criticism from his spouse through self-adjustment. Thus, the bond and closeness among family members can be well maintained.

In terms of the mediation effect of mutual communication, there is also no clear evidence verifying the influence of the mother’s gate-closing behavior on family strength (β = 0.07, *p* > 0.05) while mutual communication has a remarkably beneficial effect (β = 1.27, *p* < 0.001) and works as a complete mediation (Table 7). Even if the mother’s gate-closing behavior is perceived, timely and adequate communication could also maintain family harmony and guarantee a smooth parenting process [37,38,39]. Effective communication means more opportunities to solve problems that have emerged and enhance family cohesion. The bootstrapping results (Table 8) further verified the mediation effect of mutual communication between gate-closing behavior and family health (β = −0.37, 95% CI = −0.52~−0.24).

#### 3.2.4. Dual Mediation Analysis upon Maternal Gate Closing

**Figure 3:**
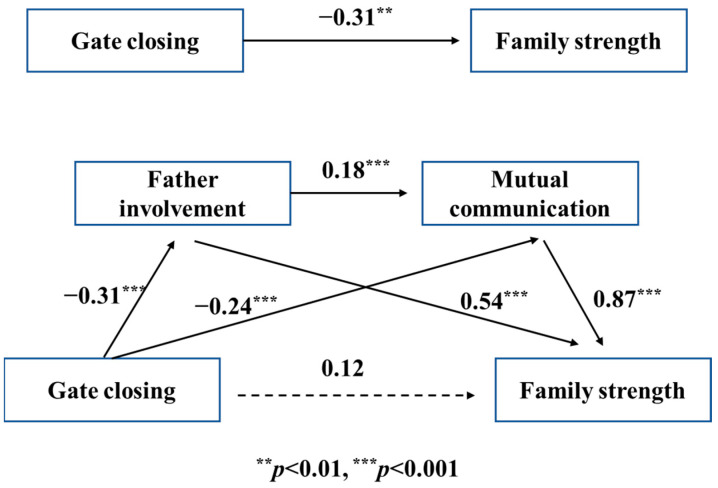
Dual mediation model of father involvement in child education and mutual communication between maternal gate closing and family strength.

As shown in Table 7 and Figure 3, SPSS PROCESS Macro Model 6 was applied in the investigation of the dual mediation effect of father involvement and mutual communication when the mother exhibits gate-closing behavior.

**Figure 4:**
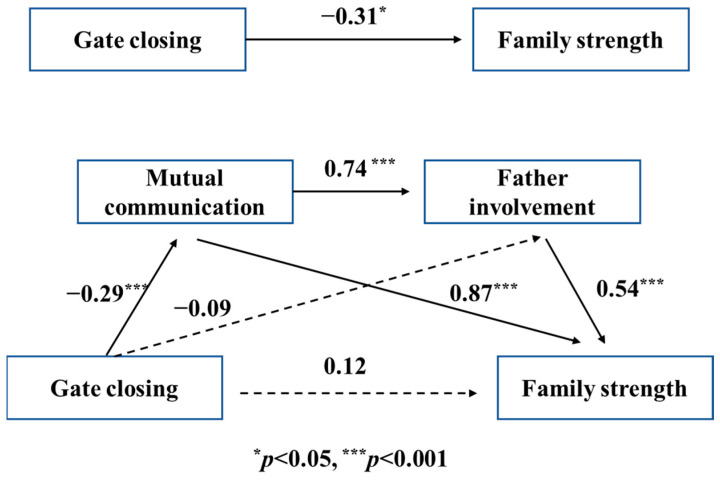
Dual mediation model of mutual communication and father involvement between maternal gate closing and family strength.

The results illustrate that gate-closing behavior has a negative effect on both family strength (β = −0.31, *p* < 0.01) and father involvement (β = −0.31, *p* < 0.001). Moreover, gate-closing behavior, father involvement, and mutual communication were analyzed together to reveal their influence on family strength; both father involvement and mutual communication have a positive correlation with family strength, while the influence of gate-closing behavior on family health is not clear (β = 0.12), demonstrating the complete dual intermediation effect of father involvement and mutual communication. The bootstrapping test results (Table 8) further confirmed the dual intermediation effect (β = −0.05, CI = −0.09~−0.02). Moreover, SPSS PROCESS Macro Model 6 illustrated that there is also a complete dual intermediation effect of mutual communication and father involvement upon mother’s gate-closing behavior (Table 7 and Figure 4), which was further confirmed in the bootstrapping analysis (β = −0.01, CI = −0.02~−0.00), as shown in Table 8.

## 4. Discussion

This research was designed to examine the impacts of maternal gatekeeping behavior on father-perceived family strength, as well as the mediation effect of mutual communication and father involvement. The results indicated that maternal gate opening behavior, father involvement, mutual communication, and family strength are positively correlated with each other, whereas maternal gate closing is negatively associated with father involvement, mutual communication, and family strength. Path analysis verified the partial (dual) mediation effect of father involvement and mutual communication between maternal gate opening and family strength, as well as a complete (dual) mediation effect upon maternal gate closing.

### 4.1. Correlation Analysis between Father-Perceived Mother Gatekeeping Behavior, Father Involvement, Mutual Communication, and Family Strength

It was found that maternal gatekeeping, father involvement, mutual communication, and family strength have significant correlations. Studies have shown that a mother’s gate-opening behavior has a positive correlation with her husband’s involvement in parenting, which suggests that encouragement and support can promote father involvement during the parenting process [40]. With sufficient encouragement and a gate-opening response, fathers generally provide more support and develop greater emotional intimacy with their family members. Conversely, if a mother exhibits gate closing attitudes, such as criticism, interception, or restriction, co-parenting may be filled with difficulties. At the same time, a mother’s gate closing behavior limits interaction between the father and child, with this gradually having a negative impact on child development [41]. As expected, this can hamper fathers’ self-esteem consolidation, leading to fewer deep conversations. Based on the crossover hypothesis, the mother’s gate-closing behavior in the parent system is harmful to pleasant parenting behavior in the father–child system to some extent [11].

A mother’s gate-opening behavior also has a positive correlation with mutual communication. Support from mothers could trigger fathers’ emotional resonance, promoting positive communication. Yu and Lee reported that there is a highly positive relationship between marital satisfaction and maternal gate-opening behavior [42]. Conversely, gate-closing behavior shows a negative relationship with communication between couples, consistent with previous research conducted by Jung and Lee [43]. Maternal interference with father involvement can harm fathers’ self-esteem to some extent, which may lead to marital conflicts and a reduction in communication.

A mother’s gate-opening behavior has a positive correlation with family strength. In this study, the significant positive effect between maternal gate-opening behavior and family strength has been unveiled. Maternal verbal and nonverbal encouragement and support for the parenting behavior of fathers is closely related to family strength [44]. On the other hand, it was found that gate closing behavior has a negative correlation with family strength. When father involvement is limited, their perception of respect and attention from their spouse is affected, which subsequently reduces their affection for their family and further weakens overall family strength.

Fathers’ involvement serves as a mediator between the mother’s gatekeeping and family strength. As suggested by Mitshell et al., mothers should better provide their spouse with sufficient opportunities to participate in the child-rearing process [45]. Fathers’ involvement has a complete mediation effect between the mother’s gate-closing behavior and family strength; thus, gate-closing behavior cannot directly decrease family strength. If fathers actively engage in the parenting process, family strength can still remain at a high level. Therefore, enhancing fathers’ parenting awareness and ability is of great value for family stability and children’s development. With the continuous evolution of the family structure, the proportion of a father’s influence on children’s physical and cognitive development is increasing. Sufficient interaction with the mother is also essential for reliable family strength.

Based on the communication theory of Koerner and coworkers, whether conceived of as a process of making facts mutually manifest or of developing and sustaining definitions of reality in relationships, communication plays a central role in the family [46]. Communication is an effective way to strengthen family bonds. When a mother exhibits gate-closing behavior, mutual communication plays a role of complete mediation. This indicates that even if a mother’s gate-closing behavior is perceived, timely and adequate communication can maintain family harmony and ensure a smooth parenting process. Effective communication provides more opportunities to solve emerging problems and enhance family cohesion. The dual mediation effect of father involvement–mutual communication has been verified in previous research [41]. However, the mediation effect of mutual communication–father involvement has not yet been identified, which means that compared to mutual communication, fathers’ parenting awareness and responsibility are more crucial. Fathers’ involvement and mutual communication have a partial (dual) mediation effect when a mother exhibits gate-opening behavior; however, there is a complete (dual) mediation effect when she exhibits gate-closing behavior. This may be related to how fathers’ parenting involvement is more easily affected by positive responses from their wives than negative ones. A father with high levels of resilience can eliminate negative effects of disapproval or criticism from his spouse through self-adjustment. Thus, the bond and closeness among family members can be maintained.

### 4.2. Significance and Limitations

To our knowledge, this is the first study to investigate maternal gatekeeping behavior from the father’s perspective, with childcare participation and mutual communication selected as mediating variables. Since the father is directly influenced by maternal gatekeeping, he has a more tangible perception of the family status; this research can effectively avoid deviations caused by the mother’s self-assessment or researchers’ observations. By systematically investigating the relationship between father-perceived family strength and maternal gatekeeping, we have gained detailed insights into the partial (dual) and complete (dual) mediation effects of father involvement and mutual communication in various gatekeeping situations.

Nevertheless, the study also has several limitations. Fathers’ involvement in parenting was measured through a self-reported questionnaire, which could be influenced by factors such as social suitability or defensive attitudes, although it was completed anonymously.

In this study, the role scale of the mother gatekeeper in Korea was used. In the future, the use of measurement tools developed according to the family situation in China can increase reliability and validity. In this study, the role of the mother gatekeeper and the mediating role of father-rearing participation and communication between couples were confirmed in the relationship between family health. It is necessary to establish various factors that affect family health and conduct continuous research on this. In this study, data were collected online through parent group chat rooms in each class. This is less reliable than the way in which they visit kindergartens directly and interview parents. Therefore, in order to receive the attention of respondents and secure responses more truthfully, future studies need to more accurately explore the relationship between variables using methods other than online surveys.

This study used the gatekeeping behavior scale developed for Korean mothers. In the future, if an instrument tool developed based on Chinese family conditions can be used, the reliability and appropriateness will be improved. This study verified the mediation effect of father involvement and mutual communication in the relationship between maternal gatekeeping behavior and family strength. It is necessary to set multiple factors that affect family strength and conduct continuous research on them. This study collected relevant information through online questionnaires, which have a lower level of reliability compared to directly interviewing parents at kindergartens. Therefore, in future studies, it is necessary to use methods other than online questionnaires to explore the relationship between variables more accurately.

### 4.3. Implications for Family Therapy and Practice

The structure of a family undergoes significant changes after the birth of a child, transforming from a simple relationship between partners to a complex family system that includes the couple system, father–child system, mother–child system, and three-part family systems. As the level of relationship complexity increases, numerous internal issues that need to be addressed exponentially increase, and the absence of any family member can lead to the collapse of family stability [47]. To ensure the participation of all members, especially the father, in family activities or work, it is highly essential for fathers to perceive encouraging maternal gate opening. This may inspire mothers to pay more attention to the effectiveness of their gate-opening behavior. Due to the increased complexity of family structures, problems or conflicts rapidly accumulate in daily life. Timely communication can avoid crises in a couple’s relationship, enhance the father’s participation in parenting, and improve family strength.

Currently, most hospitals or related social organizations provide assistance on child-rearing skills; however, their enhancement of family strength is generally limited. More professional guidance on psychological preparation for family structure change, potential family problems, and abrupt diversion of family lifestyle should be provided, as maintaining family strength is more valuable for the long-term development of children [48]. Fathers are highly recommended to make adequate preparations toward understanding the mental and physical stress of mothers and taking on responsibilities of raising children and resolving potential conflicts within the family [49] as a fully prepared father can still maintain good family strength even upon maternal gate closing.

### 4.4. Direction for Future Research

This research highlights the mediating role of father involvement and mutual communication in the relationship between maternal gatekeeping and family strength. Family strength encompasses relationship qualities that contribute to the emotional health and well-being of the family. Encouraging ongoing research on other factors that influence family strength is essential for promoting the construction of a good environment for child development. Providing sufficient and suitable support during the child’s socialization process is one of the most important functions of parents and family background. Thorough investigation of the influence of family strength, mutual communication, parent-child interaction, or maternal gatekeeping behavior on children’s emotions, cognition, and language is also essential. Further research and analysis of the father’s role in child parenting and family relationships are still highly necessary, such as establishing the relationship model between the degree of the father’s involvement and the mother’s role reversal or anxiety. Mothers play a vital role in the development of family strength. They are particularly prone to anxiety during the role reversal process. Determining the causes of anxiety and addressing them is crucial to the development of family strength, which could be an important research topic. Exceptional children with various psychological problems, such as cognitive disorders and autism spectrum disorder, generally emerge in families with low-level strength. In these families, mothers usually suffer from more psychological pressure, which may lead to unique gatekeeping tendencies. The specific maternal gatekeeping features should be further organized and analyzed with targeted family therapy proposed in future research.

### 4.5. Conclusions

In summary, the present study suggests the underlying mechanisms of father-perceived family strength influenced by maternal gatekeeping behavior. Meanwhile, the mediation effect of father involvement and marital communication also highlights the importance of parenting preparation for fathers, such as psychological self-adjustment, especially under interception or restriction.

## Figures and Tables

**Table 1:** General characteristics of the participants.

		Amount	Ratio (%)			Amount	Ratio (%)
Age (child)	3 years	110	34.4	Age (father)	<30	52	16.3
	4 years	102	31.9		30–40	240	75.0
	5 years	108	33.8		>40	28	8.8
Gender (child)	Male	184	57.5	Education level (father)	Junior school education	12	3.8
	Female	136	42.5		High school education	40	12.5
Total		320	100.0		College degree	68	21.3
		**Amount**	**Ratio (%)**		Bachelor	128	40.0
Average monthly income (father)	<3000 RMB	20	6.3		Master/Ph.D.	72	22.5
	3000–less than 6000 RMB	92	30.6	Occupation (father)	Government employee	18	5.6
	6000–less than 10,000 RMB	112	35.0		Businessman	30	9.4
	≥10,000 RMB	90	28.1		Technical Position	60	18.8
Number of children (father)	1	192	60.0		Service sector	10	3.1
	2	124	38.8		White-collar worker	108	33.8
	3	4	1.3		Blue-collar worker	62	19.4
	>3	0	0.0		Others	32	10.0
Total		320	100.0	Total		320	100.0

**Table 2:** Measurement of family strength, maternal gatekeeping, and father involvement in child education.

	Sub-Factors	NI	QI	C (α)		Sub-Factors	NI	QI	C (α)
Family strength	Dedication and care	7	1–7	0.857	Father involvement in child education	Rule guidance	3	1–3	0.727
	Family resilience	7	8–14	0.871		Kindergarten life support	3	4–6	0.735
	Mutual respect and tolerance	5	15–19	0.786		Mother assistance	3	7–9	0.820
	Love expression and sharing	4	20–23	0.843		Childcare support	3	10–12	0.616
	Total	23		0.834		Company and communication	3	13–15	0.824
Maternal gatekeeping	Gate opening	9	1–9	0.908		Encouragement and praise	3	16–18	0.831
	Gate closing	8	10–17	0.785		Total	18		0.884

NI: number of items; QI: question item; C (α): Cronbach’s α value.

**Table 3:** The impact of demographic factors on family strength.

		Amount	Family Strength			
			M (SD)	t/F	*p*	Scheffé
Age (father)	<30	52	98.31 (14.72)	2.20	0.112	
	30–40	240	96.09 (12.16)			
	>40	28	92.14 (11.20)			
Education level (father)	Junior school education	12	91.50 (14.51)	0.98	0.421	
	High school education	40	97.35 (11.15)			
	College degree	68	97.76 (12.80)			
	Bachelor	128	95.97 (13.57)			
	Master/Ph.D.	72	94.86 (10.82)			
Occupation (father)	Government employee	18	90.22 (14.63)	3.12	0.053	
	Businessman	30	97.80 (7.45)			
	Technical Position	60	93.37 (12.84)			
	Service sector	10	86.00 (17.04)			
	White-collar worker	108	97.56 (11.34)			
	Blue-collar worker	62	96.94 (13.72)			
	Others	32	99.63 (12.42)			
Average monthly income (father)	<3000 RMB	20	95.80 (17.64)	2.92	0.045 *	b < d
	3000–less than 6000 RMB	98	93.33 (13.95)			
	6000–less than 10,000 RMB	112	96.55 (11.07)			
	≥10,000 RMB	90	98.64 (10.97)			
Number of children (father)	1	192	96.16 (13.54)	0.02	0.990	
	2	124	96.00 (10.72)			
	3	4	97.00 (20.79)			

* *p* < 0.05; M: mean value; SD: standard deviation.

**Table 4:** Correlation between maternal gatekeeping, family strength, father involvement, and mutual communication during the child education process.

	1	1-1	1-2	1-3	1-4	2-1	2-2	3	3-1	3-2	3-3	3-4	3-5	3-6	4
1. Family strength	1														
1-1. Dedication and care	0.866 ***	1													
1-2. Flexibility and adaptability	0.903 ***	0.701 ***	1												
1-3. Respect and tolerance	0.865 ***	0.643 ***	0.703 ***	1											
1-4. Love and share	0.859 ***	0.687 ***	0.700 ***	0.677 ***	1										
2-1. Gate opening	0.681 ***	0.630 ***	0.520 ***	0.611 ***	0.649 ***	1									
2-2. Gate closing	−0.171 **	−0.243 ***	−0.066	−0.174 **	−0.127 *	−0.188 **	1								
3. Father involvement	0.554 ***	0.498 ***	0.451 ***	0.476 ***	0.476 ***	0.621 ***	−0.222 ***	1							
3-1. Rule guidance	0.451 ***	0.438 ***	0.388 ***	0.373 ***	0.381 ***	0.498 ***	−0.170 **	0.762 ***	1						
3-2. Kindergarten life support	0.336 ***	0.297 ***	0.273 ***	0.286 ***	0.332 ***	0.447 ***	−0.071	0.789 ***	0.542 ***	1					
3-3. Mother assistance	0.542 ***	0.483 ***	0.465 ***	0.454 ***	0.504 ***	0.599 ***	−0.235 ***	0.825 ***	0.588 ***	0.574 ***	1				
3-4. Childcare support	0.428 ***	0.411 ***	0.335 ***	0.374 ***	0.392 ***	0.436 ***	−0.239 ***	0.760 ***	0.430 ***	0.483 ***	0.561 ***	1			
3-5. Companion and talk	0.464 ***	0.416 ***	0.370 ***	0.389 ***	0.470 ***	0.524 ***	−0.152 **	0.841 ***	0.593 ***	0.590 ***	0.601 ***	0.607 ***	1		
3-6. Encourage and praise	0.432 ***	0.342 ***	0.328 ***	0.405 ***	0.405 ***	0.462 ***	−0.209 ***	0.804 ***	0.457 ***	0.563 ***	0.626 ***	0.621 ***	0.600 ***	1	
4. Mutual communication	0.502 ***	0.491 ***	0.394 ***	0.450 ***	0.450 ***	0.503 ***	−0.400 ***	0.412 ***	0.305 ***	0.320 ***	0.408 ***	0.312 ***	0.312 ***	0.317 ***	1

* *p* < 0.05, ** *p* < 0.01, *** *p* < 0.001.

**Table 5:** Mediation effect and dual mediation effect of father involvement and mutual communication between maternal gate opening and family strength.

	Pathway	β	SE	T	F	R^2^	LLCI	ULCI
Mediation effect (father involvement)	X→M1	0.81	0.06	14.14 ***	200.05 ***	0.39	0.70	0.93
	X→Y	1.15	0.07	16.59 ***	275.37 ***	0.46	1.01	1.28
	X→Y M1→Y	0.92 0.27	0.09 0.07	10.74 *** 4.18 ***	153.55 ***	0.49	0.75 0.15	1.09 0.40
Mediation effect (mutual communication)	X→M2	0.34	0.03	10.38 ***	107.67 ***	0.25	0.28	0.41
	X→Y	1.15	0.07	16.59 ***	275.37 ***	0.46	1.01	1.26
	X→Y M2→Y	0.97 0.52	0.08 0.11	12.47 *** 4.63 ***	79.90 ***	0.50	0.81 0.30	1.12 0.75
Dual mediation effect (M1→M2)	X→Y	1.15	0.07	16.60 ***	275.37 ***	0.46	1.01	1.28
	X→M1	0.81	0.06	14.14 ***	200.05 ***	0.39	0.70	0.93
	X→M2 M1→M2	0.28 0.06	0.04 0.03	6.56 *** 2.65 ***	58.37 ***	0.27	0.19 0.02	0.36 0.15
	X→Y M1→Y M2→Y	0.79 0.23 0.46	0.09 0.06 0.11	8.90 *** 3.63 *** 4.13 ***	113.23 ***	0.52	0.62 0.11 0.24	0.97 0.36 0.68
Dual mediation effect (M2→M1)	X→Y	1.15	0.10	11.70 ***	136.82 ***	0.46	0.95	1.34
	X→M2	0.34	0.05	7.31 ***	53.50 ***	0.25	0.25	0.44
	X→M1 M2→M1	0.73 0.26	0.09 0.14	7.75 *** 1.87	58.37 ***	0.40	0.54 −0.02	0.91 0.53
	X→Y M2→Y M1→Y	0.79 0.46 0.23	0.13 0.16 0.09	6.25 *** 2.90 ** 2.55 *	55.90 ***	0.52	0.54 0.15 0.05	1.05 0.78 0.42

* p < 0.05, ** *p* < 0.01, *** *p* < 0.001; X: maternal gatekeeping; Y: family strength; M1: father involvement; M2: mutual communication; SE: standard error; CI: confidence interval; LLCI: lower limit confidence interval; ULCI: upper limit confidence interval.

**Table 6:** Bootstrapping analysis of the mediation effect and dual mediation effect upon gate opening.

Mediation Effect (Father Parenting Participation)					Mediation Effect (Mutual Communication)				
Effect	β	Boot SE	95% confidence interval		Effect	β	Boot SE	95% confidence interval	
			Boot LLCI	Boot ULCI				Boot LLCI	Boot ULCI
Indirect effect	0.22	0.07	0.10	0.36	Indirect effect	0.18	0.05	0.09	0.29
Direct effect	0.92	0.09	0.74	1.11	Direct effect	0.52	0.13	0.28	0.80
Total effect	1.15	0.06	1.03	1.27	Total effect	1.15	0.06	1.03	1.27
**Dual Mediation effect (M1→M2)**					**Dual Mediation effect (M2→M1)**				
**Indirect** **effect**	**β**	**Boot SE**	**95% confidence interval**		**Indirect** **effect**	**β**	**Boot SE**	**95% confidence interval**	
			**Boot LLCI**	**Boot ULCI**				**Boot LLCI**	**Boot ULCI**
Ind1 X→M1→Y	0.19	0.06	0.07	0.33	Ind1 X→M2→Y	0.16	0.07	0.04	0.32
Ind2 X→M2→Y	0.13	0.04	0.06	0.22	Ind2 X→M1→Y	0.17	0.09	0.02	0.35
Ind3 X→M1→M2→Y	0.03	0.02	0.01	0.07	Ind3 X→M2→M1→Y	0.02	0.02	−0.00	0.06
Total	0.35	0.07	0.21	0.50	Total	0.35	0.10	0.16	0.56

X: maternal gatekeeping; Y: family strength; M1: father involvement; M2: mutual communication; SE: standard error; CI: confidence interval; LLCI: lower limit confidence interval; ULCI: upper limit confidence interval.

**Table 7:** Mediation effect and dual mediation effect of father involvement and mutual communication on maternal gate closing and family strength during the child education process.

	Pathway	β	SE	t	F	R^2^	LLCI	ULCI
Mediation effect (father involvement)	X→M1	−0.31	0.08	−4.05 ***	16.41 ***	0.05	−0.46	−0.16
	X→Y	−0.31	0.10	−3.09 **	9.55 **	0.03	−0.50	−0.11
	X→Y M1→Y	−0.09 0.70	0.09 0.06	−1.05 11.35 ***	71.13 ***	0.31	−0.26 0.58	0.08 0.82
Mediation effect (mutual communication)	X→M2	−0.29	0.04	−7.79 ***	60.72 ***	0.16	−0.37	−0.22
	X→Y	−0.31	0.10	−3.09 **	9.55 **	0.03	−0.51	−0.11
	X→Y M2→Y	0.07 1.27	0.10 0.13	0.68 9.74 ***	53.64 ***	0.25	−0.12 1.01	0.25 1.52
Dual mediation effect (M1→M2)	X→Y	−0.31	0.10	−3.09 **	9.55 **	0.03	−0.51	−0.11
	X→M1	−0.31	0.08	−4.05 ***	16.41 ***	0.05	−0.46	−0.16
	X→M2 M1→M2	−0.24 0.18	0.04 0.03	−0.61 *** 6.91 ***	58.74 ***	0.27	−0.31 0.13	−0.17 0.23
	X→Y M1→Y M2→Y	0.12 0.54 0.87	0.09 0.06 0.13	1.35 8.83 *** 6.93 ***	70.44 ***	0.41	−0.05 0.42 0.62	0.29 0.66 1.11
Dual mediation effect (M2→M1)	X→Y	−0.31	0.14	−2.18 *	4.74 *	0.03	−0.59	−0.03
	X→M2	−0.29	0.05	−5.49 ***	30.17 ***	0.16	−0.40	−0.19
	X→M1 M2→M1	−0.09 0.74	0.11 0.15	−0.85 4.87 ***	16.50 ***	0.17	−0.31 0.44	0.13 1.04
	X→Y M2→Y M1→Y	0.12 0.87 0.54	0.12 0.18 0.09	0.95 4.87 *** 6.20 ***	34.78 ***	0.40	−0.13 0.52 0.37	0.36 1.22 0.72

* *p* < 0.05, ** *p* < 0.01, *** *p* < 0.001; X: maternal gatekeeping; Y: family strength; M1: father involvement; M2: mutual communication; SE: standard error; CI: confidence interval; LLCI: lower limit confidence interval; ULCI: upper limit confidence interval.

**Table 8:** Bootstrapping analysis of the mediation effect and dual mediation effect upon gate closing.

Mediation Effect (Father Parenting Participation)					Mediation Effect (Mutual Communication)				
Effect	β	Boot SE	95% confidence interval		Effect	β	Boot SE	95% confidence interval	
			Boot LLCI	Boot ULCI				Boot LLCI	Boot ULCI
Indirect effect	−0.22	0.06	−0.35	−0.10	Indirect effect	−0.37	0.07	−0.52	−0.24
Direct effect	−0.09	0.11	−0.31	0.11	Direct effect	0.07	0.11	−0.17	0.27
Total effect	−0.31	0.13	−0.57	−0.08	Total effect	−0.31	0.13	−0.57	−0.08
**Dual mediation effect (M1→M2)**					**Dual mediation effect (M2→M1)**				
**Indirect** **effect**	**β**	**Boot SE**	**95% confidence interval**		**Indirect** **effect**	**β**	**Boot SE**	**95% confidence interval**	
			**Boot LLCI**	**Boot ULCI**				**Boot LLCI**	**Boot ULCI**
Ind1 X→M1→Y	−0.17	0.05	−0.28	−0.08	Ind1 X→M2→Y	−0.02	0.01	−0.03	−0.01
Ind2 X→M2→Y	−0.21	0.05	−0.31	−0.11	Ind2 X→M1→Y	−0.00	0.01	−0.02	0.01
Ind3 X→M1→M2→Y	−0.05	0.02	−0.09	−0.02	Ind3 X→M2→M1→Y	−0.01	0.00	−0.02	−0.00
Total	−0.43	0.08	−0.58	−0.28	Total	−0.04	0.01	−0.05	−0.02

X: maternal gatekeeping; Y: family strength; M1: father involvement; M2: mutual communication; SE: standard error; CI: confidence interval; LLCI: lower limit confidence interval; ULCI: upper limit confidence interval.

## Data Availability

The data presented in this study are available on request from the corresponding author. The data are not publicly available due to the requests of participants and related confidentiality agreement.

## Supplementary Materials

The following supporting information can be downloaded at: https://www.mdpi.com/article/10.3390/bs13120968/s1, File S1: Checklist used for manuscript preparation and Investigation questionnaire.
