# Supplementary material for: The Relations between Father-Perceived Family Strength and Maternal Gatekeeping in Chinese Families—Focusing on the Dual Mediation Effect of Father Involvement and Mutual Communication
Source: Behav Sci (Basel). 2023 Nov 24;13(12):968. doi: 10.3390/bs13120968 (PMC10740795; doi:10.3390/bs13120968)
Supplement: Supplementary file 1 [file behavsci-13-00968-s001.zip › behavsci-2626561-supplementary.pdf]

Supplementary Materials

The Relations between Father-Perceived Family Strength and Maternal Gatekeeping in Chinese Families—Focusing on the Dual Mediation Effect of Father Involvement and Mutual Communication

Shu Zhang [1,2,*] and Hae-Shin Hwang [1,*]

[1] Department of Family Welfare, Sangmyung University, Seoul 04763, Republic of Korea
[2] West Campus, Shandong University of Technology, Zibo 255000, China
* Correspondence: zhangshu@sdut.edu.cn (S.Z.); hshwang@smu.ac.kr (H.-S.H.)

Table S1. Checklist used for manuscript preparation.

Topic		Number	Description
Title and Abstract		1	1. Describing the research type using professional terminology.<br>2. Comprehensive abstract that accurately describes the research methods and results while explaining the scientific background and rationale.
Introduction	Background and rationality	2	Interpreting the scientific background and basis of the study.
	Research objectives	3	Expounding the research objectives.
Methodology	Research object	4	1. Describing the characteristics of the study subjects and describing the follow-up methods.<br>2. Discussing typical important aspects, such as the eligibility criteria for the participants, potential confounding factors, and potential sources of bias.
	Research instrument	5	1. Detailed description of the development, history, and application methods of each research instrument.<br>2. Describing the content of the research scale and related items.
	Analysis method	6	After data collection, the analytical methods are described in detail.
Results	Main results	7	1. Providing the association values between different research elements.<br>2. Their related precision value is also calculated.
Discussion	Important discovery	8	Summarizing the significant findings related to the research hypotheses.
	Significance /limitations	9	Discussing the significance of the research and potential limitations.
	Implications	10	Discussing the generalizability and applicability of the research results.

Investigation Questionnaire

Dear Fathers,

Hello, I am a graduate student majoring in child development and education at Sangmyung University in South Korea. I am currently conducting a study on families with children aged 3–5. The purpose of this study is to investigate the relationship between fathers’ perception of mothers’ gatekeeping behavior and family strength and to collect research data. In order to fully understand the connection, please complete the questionnaire carefully. This questionnaire consists of five scales, all completed by the child's father; you only need to tick the options that match your actual situation.
All information provided by you in the survey will only be processed statistically; please rest assured that no personal information will be involved. Your honest completion of this questionnaire is very important for our research. Thank you for your help.

Part 1: Basic Information

I. Basic Information of the Father (the following "you" refers to the father himself)

1. What is your age?
① Under 30 years old ② 30~40 years old ③ Over 40 years old
2. What is your highest degree?
① High school or lower ② Secondary vocational school or high school ③ Junior college ④ Undergraduate ⑤ Postgraduate or above
3. What is your occupation?
① National civil servant ② Self-employed business owner ③ Cultural, educational, scientific, medical, and legal personnel (such as teachers, doctors, lawyers, etc.) ④ Business and service personnel ⑤ Company clerk ⑥ Worker/manual laborer ⑦ Other
4. What is your monthly income?
① Less than 3000 yuan ② 3000~6000 yuan ③ 6000~10000 yuan ④ More than 10000 yuan

II. Basic Information of the Child (the child currently attending kindergarten)

1. Gender of the child:
① Male ② Female
2. Age of the child:
① 3 years old ② 4 years old ③ 5 years old
3. Total number of children:
① One child ② Two children ③ Three or more children

III. Family structure:

① Single-parent family ② You and your partner live with your child together ③ You and your partner live with your parents and children in three generations

Part 2: Research Instruments

2-1. Family Strength

Questionnaire to be filled out by the father; please choose the option that best matches your actual situation and place a tick in the corresponding box.

Question	Never	Occasionally	Sometimes	Often	Always
Dedication and care					
1. Family members trust each other					
2. Respect for the parents and taking care of them					
3. Mutual consideration and care between couples and two generations					
4. Achievements in family efforts are recognized and appreciated					
5. Family members are honest and trustworthy					
6. Detect changes in family members' emotions and provide support and care when necessary					
7. Family members can fairly and reasonably share family obligations and responsibilities					
Family resilience					
8. Personal choices made by family members can be respected by others					
9. The rules or habits previously established in the family will change according to new situations					
10. Parents/elders can put down their pride and learn from their children/younger generation or correct their mistakes when they are wrong					
11. No matter what troubles arise, family members believe that things will get better					
12. When facing new problems or situations, our family has flexible adaptability					
13. When encountering difficulties or pressures, family members can provide effective help and support					

14. For major events in the future of the family, we make plans in advance and actively prepare for them					
Mutual respect and tolerance					
15. Can freely and equally express opinions when making important family decisions					
16. When there are different opinions and disagreements, everyone can be humble and compromise					
17. Rarely criticize or blame each other among family members					
18. Never say hurtful words or do harmful things to each other, even when angry					
19. Never resort to physical violence or hitting each other under any circumstances					
Love expression and share					
20. Often express love through language or actions among family members					
21. Try to spend time with family members as much as possible					
22. Often laugh heartily and feel relaxed and happy when family members are together					
23. Family life is not boring, often with small touches of romance and freshness					

2-2. Maternal Gatekeeping

Questionnaire to be filled out by the father; please choose the option that best matches your actual situation and place a tick in the corresponding box.

Question	Never	Rarely	A few times	Occasionally	Often	Always
Gate opening						
1. My wife seeks my help in the process of parenting and child-rearing						
2. My wife expresses gratitude for my contributions and help in the process of parenting and child-rearing						
3. My wife praises me for being a good father						
4. My wife consults me in the process of parenting and child-rearing						
5. My wife praises me in front of others for being a good father						

6. My wife praises me in front of our children for providing them with enough sense of security and happiness						
7. My wife encourages me to spend time alone with our children						
8. My wife respects my parenting methods and approaches						
9. My wife values my parenting plans or ideas						
Gate closing						
10. My wife criticizes my parenting methods						
11. My wife expresses dissatisfaction when she disagrees with my parenting methods						
12. My wife complains about my parenting behavior to others						
13. When I adopt inappropriate parenting methods, my wife intervenes with her own correct methods on the spot						
14. My wife criticizes my inappropriate parenting methods and behavior in front of our children						
15. Although my wife does not show dissatisfaction with the housework I do in front of her, she will redo it after I leave						
16. My wife's overall level of parenting and education is higher than mine						
17. In the process of parenting and child-rearing, my wife will not seek my help because she does not trust my parenting behavior and methods						

2-3. Mutual communication

Questionnaire to be filled out by the father; please choose the option that best matches your actual situation and place a tick in the corresponding box.

Question	Never	Rarely	Occasionally	Often	Always
1. It is very easy for me to express my true feelings to my spouse					
2. When there are conflicts between us, my spouse often remains silent					
3. My spouse sometimes expresses some derogatory opinions about me					
4. Sometimes, I don't dare ask my spouse for what I need					
5. I hope my spouse shares his/her feelings with me					

6. Sometimes, it's hard for me to believe everything my spouse tells me					
7. I often don't tell my spouse how I feel because she should be able to understand on her own					
8. We are very satisfied with the way we communicate with each other as a couple					
9. I don't always share my troubles with my spouse because I'm afraid of her temper					
10. My spouse always listens carefully when I speak					

2-4. Father involvement

Questionnaire to be filled out by the father; please choose the option that best matches your actual situation and place a tick in the corresponding box.

Question	Never	Rarely	Occasionally	Often	Always
1. Encourage and guide children to tidy up their own belongings					
2. Set rules and boundaries for children's behavior					
3. Encourage and help children to complete a task from beginning to end					
4. Praise and compliment children when they show off their works or good deeds completed in kindergarten					
5. Assist children in completing tasks assigned by kindergarten teachers					
6. Join in activities that children participate in					
7. Give encouragement and emotional support to the child's mother					
8. Let the child know the importance of him/her and his/her mother					
9. Help the child's mother raise the child together					
10. Meet the child's basic material needs					
11. Take care of the child's daily life (such as feeding, dressing, and transporting to kindergarten)					
12. Take responsibility for providing financial support for the child					
13. When the child wants to talk, take time to talk with him/her					
14. Take time to accompany the child to do what he/she wants to do					
15. Do housework with the child					
16. Praise the child for doing something well or correctly					
17. Praise the child for learning to persevere					
18. Tell the child that you love him/her (such as touching the head, hugging, or kissing)					
